# Copper Nanoclusters Anchored on Crumpled N-Doped MXene for Ultra-Sensitive Electrochemical Sensing

**DOI:** 10.3390/s25082508

**Published:** 2025-04-16

**Authors:** Hanxue Yang, Chao Rong, Shundong Ge, Tao Wang, Bowei Zhang, Fu-Zhen Xuan

**Affiliations:** 1 Shanghai Key Laboratory of Intelligent Sensing and Detection Technology, East China University of Science and Technology, Shanghai 200237, China; y30220691@mail.ecust.edu.cn (H.Y.); y10240088@mail.ecust.edu.cn (C.R.); geshundong@mail.ecust.edu.cn (S.G.); 2 Key Laboratory of Pressure Systems and Safety of Ministry of Education, East China University of Science and Technology, Shanghai 200237, China; 3 School of Mechanical and Power Engineering, East China University of Science and Technology, Shanghai 200237, China

**Keywords:** MXene, Cu nanoclusters, crumpled structure, dopamine, uric acid

## Abstract

**Highlights:**

**What are the main findings?**

1. 3D crumpled MXene with strain wrinkles enhances conductivity and prevents restacking.
2. Cu nanoclusters (3.0 wt%) synergize N-doping for noble metal-like catalytic activity.

**What is the implication of the main finding?**

1. Ultrasensitive dual-analyte non-enzymatic detection of DA/UA with anti-interference capability and rapid response.
2. Novel material design strategies provide new approaches for the development of future high-performance sensors.

**Abstract:**

Simultaneous detection of dopamine (DA) and uric acid (UA) is essential for diagnosing neurological and metabolic diseases but hindered by overlapping electrochemical signals. We present an ultrasensitive electrochemical sensor using copper nanoclusters anchored on nitrogen-doped crumpled Ti_3_C_2_T_x_ MXene (Cu-N/Ti_3_C_2_T_x_). The engineered 3D crumpled architecture prevents MXene restacking, exposes active sites, and enhances ion transport, while Cu nanoclusters boost electrocatalytic activity via accelerated electron transfer. Structural analyses confirm uniform Cu dispersion (3.0 wt%), Ti-N bonding, and strain-induced wrinkles, synergistically improving conductivity. The sensor achieves exceptional sensitivity (1958.3 and 1152.7 μA·mM^−1^·cm^−2^ for DA/UA), ultralow detection limits (0.058 and 0.099 μM for DA/UA), rapid response (<1.5 s), and interference resistance (e.g., ascorbic acid). Differential pulse voltammetry enables independent linear detection ranges (DA: 2–60 μM; UA: 5–100 μM) in biofluids, with 94.4% stability retention over 7 days. The designed sensor exhibits excellent capabilities for DA and UA detection. This work provides a novel design strategy for developing high-performance electrochemical sensors.

## 1. Introduction

Dopamine (DA) and uric acid (UA) are vital biomarkers for disease diagnosis [1]. DA, a neurotransmitter linked to psychiatric diseases, regulates neurological functions [2,3]. UA, a purine metabolite, protects vascular health but induces gout and cardiovascular disorders at high concentrations [4,5,6]. Their coexistence in biological fluids and correlated concentration changes (e.g., reduced DA with elevated UA in Parkinson’s disease) underscore the need for simultaneous detection [7]. However, accurate detection is hampered by the similarity of the signals of DA and UA and the presence of other interferences during the detection process [8]. Developing high-performance sensors is crucial for advancing clinical diagnostics and mechanistic studies [9]. Current methods for DA/UA detection, such as high-performance liquid chromatography-mass spectrometry (HPLC-MS) and enzyme-linked immunosorbent assays (ELISA), face limitations such as high cost, lengthy procedures, and poor portability, limiting their clinical utility [10,11,12,13,14]. Electrochemical sensing emerges as a promising alternative due to rapid response, cost efficiency, multi-target capability, and portability, making it ideal for clinical diagnostics and dynamic disease studies [15,16,17]. However, existing sensing materials (carbon nanomaterials, MOFs, etc.) suffer from narrow detection ranges, low sensitivity, high detection limits, and poor selectivity against interferents like ascorbic acid [18,19,20,21,22]. To address these challenges, designing advanced nanomaterials with enhanced selectivity, sensitivity, and ultralow detection limits is crucial for enabling real-time clinical monitoring and precise disease progression analysis.

Transition metal carbide/nitride (MXene) is a diverse family of two-dimensional (2D) nanomaterials with excellent electrical conductivity, outstanding sensing performance, tunable surface chemistry, and layered structures, which have versatile applications in energy and sensing fields [23,24]. Ti_3_C_2_T_x_ MXene demonstrates exceptional electrochemical sensing capabilities due to its high conductivity and rich surface functional groups [25,26,27]. However, van der Waals-driven restacking of nanosheets reduces active site exposure and ion diffusion efficiency [28]. To address this, constructing a 3D crumpled structure through strain engineering effectively inhibits stacking, creating hierarchical pores and interconnected channels that shorten ion diffusion distances and enhance interfacial electron transfer [29,30,31]. Existing studies have shown that Cu nanoclusters have good sensing performance [32,33]. But the poor stability of Cu nanoclusters and the concomitant formation of Cu_2_O nanoparticles while anchoring Cu NCs limit the high atomic utilization efficiency of Cu NCs, affecting their sensitivity [34,35,36,37]. The crumpled surface of MXene can provide abundant anchor sites for loading Cu NCs and enhance the electrochemical catalytic performance through metal-carrier synergy [38,39,40]. Therefore, anchoring non-precious metal nanoclusters on crumpled MXene can achieve ultra-sensitive detection with noble metal-like properties at a low cost.

In this work, we loaded non-precious metal Cu nanoclusters onto crumpled N-doped Ti_3_C_2_T_x_ nanosheets (Cu-N/Ti_3_C_2_T_x_) and used them for the electrochemical sensing detection of DA and UA. The introduction of surface-strained crumpled structures and anchoring of Cu nanoclusters not only enhanced material stability, created a more active specific surface area, and provided additional reactive sites, but also improved electrical conductivity and electrochemical performance. The Cu-N/Ti_3_C_2_T_x_ modified electrodes exhibited high sensitivity, superior selectivity, and rapid response towards DA and UA as tested by cyclic voltammetry (CV) and differential pulse voltammetry (DPV) experiments.

## 2. Experimental Section

### 2.1. Materials

Melamine (C_3_H_6_N_6_, ≥99%), Copper (II) chloride dihydrate (CuCl_2_·2H_2_O, ≥99.0%), Lithium fluoride (LiF, 99.99%) and Hydrochloric acid (HCl, 36.0~38.0%) were supplied by Macklin. Nafion solution (5%), ascorbic acid (AA, ≥99.0%), dopamine (DA, 98%), and uric acid (UA, 99%) were supplied by Aladdin. Phosphate-buffered saline (PBS, PH = 7.4, 0.1 M) was bought from Shanghai Yuanye Bio-Technology Co., Ltd. (Shanghai, China). Ti_3_AlC_2_ MAX (400 mesh, 99.5 wt.%) was bought from Foshan XinXi Technology Co., Ltd. (Foshan, China). Argon (Ar, 99.99%) was obtained from Shanghai HaoQi Gas Co., Ltd. (Shanghai, China).

### 2.2. Synthesis of Monolayer Ti_3_C_2_T_x_ Nanosheets

Ti_3_C_2_T_x_ MXene was obtained by etching away the Al layer of Ti_3_AlC_2_ MAX using hydrogen fluoride (HF). Firstly, HF was prepared by adding 1.6 g of LiF to 9 M 20 mL of HCl and stirring for 15 min to fully dissolve. Next, 1 g of Ti_3_AlC_2_ MAX phase was slowly added and stirred in a water bath at 40 °C for 48 h. The etchant was rinsed twice with 1 M HCl and then centrifuged several times (1 min each time) with deionized water (DIW) at 5000 rpm at high speed until the supernatant did not appear in the centrifuge tube after a certain number of centrifugations. When the liquid supernatant was blackened, the tube was shaken well, and centrifugation was continued, repeating the above steps one to three times. Next, pass Ar gas, sonicate in an ice bath for 30 min, and centrifuge at 3500 rpm for 30 min to obtain a monolayer Ti_3_C_2_T_x_ MXene colloidal solution. After suction filtration of the colloid for several hours, the obtained Ti_3_C_2_T_x_ MXene film was dried in a vacuum drying oven at 60 °C for 12 h, and finally the monolayer Ti_3_C_2_T_x_ MXene solid was obtained.

### 2.3. Synthesis of Cu-N/Ti_3_C_2_T_x_, N/Ti_3_C_2_T_x_, and Cu-Ti_3_C_2_T_x_ Nanosheets

Using ice bath sonication and stirring, 60 mg of the prepared monolayer Ti_3_C_2_T_x_ MXene was completely dissolved into 15 mL DIW. 180 mg of protonated melamine was taken and completely dissolved into 0.1 M 50 mL hydrochloric acid. Then, the two mixes were stirred continuously for 1 h. 3 mg of CuCl_2_·2H_2_O was added and stirred for 1 h. The obtained mixed precursor was freeze-dried for 72 h and then placed in a tube furnace and annealed with argon gas at 400 °C for 2 h. The final Cu-N/Ti_3_C_2_T_x_ sample was obtained.

The same prepared procedure of Cu/N-Ti_3_C_2_T_x_ was conducted for the synthesis of N/Ti_3_C_2_T_x_ without the addition of metal precursors. For Cu-Ti_3_C_2_T_x_, the same steps are synthesized without adding positively charged melamine.

### 2.4. Preparation of Three Modified Electrodes

The prepared three materials were dispersed in DIW and shaken well. Next, the preparation of the modified electrode was completed by dropping 12 μL of the solution onto the glassy carbon electrode (GCE, φ = 3 mm).

### 2.5. Material Characterizations and Electrochemical Measurements

The morphology and microstructure of the materials were investigated using field emission scanning electron microscopy (FESEM) (JSM-IT800HL, Jeol, Tokyo, Japan). The chemical structure and elemental composition of the materials were further analyzed with X-ray diffractometer (XRD) (Cu-Kα radiation source, λ = 1.54059 Å, Rigaku D/max 2550VB/PC), X-ray Photoelectron Spectroscopy (XPS) (ESCALAB 250Xi, Thermo Fisher Scientific-CN, Shanghai, China), and the atomic-resolution high-angle annular dark-field scanning transmission electron microscopy (HAADF-STEM) (200 kV ThermoFisher Talos F200 X FETEM, Thermo Fisher, Waltham, MA, USA). Energy-dispersive X-ray spectroscopy (EDS) was performed using four array super X detectors. The copper loading in the material was determined using inductively coupled plasma atomic emission spectroscopy (ICP-AES, Agilent, 167–785 nm/725, Santa Clara, CA, USA).

Complete all electrochemical testing experiments with the CHI660E electrochemical workstation and three-electrode system. In which glassy carbon electrodes modified with Cu-N/Ti_3_C_2_T_x_, N/Ti_3_C_2_T_x_, and Cu-Ti_3_C_2_T_x_ were used as the working electrode, while the counter and reference electrodes were Pt wire and Ag/AgCl chloride reference electrodes, respectively. Electrochemical tests were completed by dissolving DA and UA using 0.1 M PBS (pH = 7.4).

## 3. Results and Discussion

### 3.1. Characterization of Cu-N/Ti_3_C_2_T_x_

**Figure 1:**
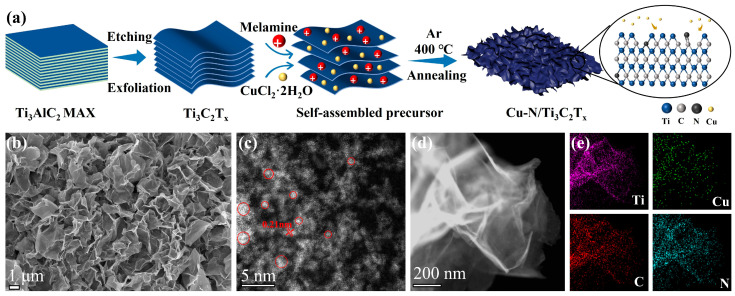
Schematic synthesis process and microstructure characterizations of Cu-N/Ti_3_C_2_T_x_. (**a**) Schematic synthesis of Cu-N/Ti_3_C_2_T_x_. (**b**) SEM image. (**c**) Atomic-resolution HAADF-STEM image (the red circles mark the Cu nanoclusters). (**d**) HAADF-STEM image and (**e**) EDS mapping images (Ti, purple; Cu, green; C, red; N, blue) of Cu-N/Ti_3_C_2_T_x_.

The synthesis of crumpled N-doped Cu nanoclusters-modified Ti_3_C_2_T_x_ MXene was carried out in a three-step process, as shown in Figure 1a. Firstly, the Al layer in the Ti_3_AlC_2_ MAX phase was selectively etched away by HF generated by mixing LiF/HCl, and an oligo- or monolayer Ti_3_C_2_T_x_ colloidal suspension was formed by ultrasonication and high-speed centrifugation [28,41]. Subsequently, CuCl_2_·2H_2_O was added to the Ti_3_C_2_T_x_ suspension. Attributed to the presence of two low-valence Ti species (Ti^2+^/Ti^3+^) of Ti_3_C_2_T_x_, a self-reduction process of copper (II) ions occurred during continuous stirring [42,43,44]. Due to the negative charge of Ti_3_C_2_T_x_ MXene, the added protonated melamine, which acts as a nitrogen source and separator, adsorbed onto MXene nanosheets to undergo electrostatic self-assembly [45]. The precursor was freeze-dried and then annealed at high temperature. And the thermal decomposition of melamine could introduce N doping and result in a crumpled structure to avoid the stacking of MXene, which finally leads to obtaining Cu-N/Ti_3_C_2_T_x_. As a comparison experiment, only CuCl_2_·2H_2_O and protonated melamine were added to the Ti_3_C_2_T_x_, respectively, and after the same treatment, Cu cluster-modified Ti_3_C_2_T_x_ (Cu-Ti_3_C_2_T_x_) and surface strain-modulated wrinkled N-doped Ti_3_C_2_T_x_ (N/Ti_3_C_2_T_x_) were obtained by annealing.

The synthetic materials were characterized using an X-ray diffractometer (XRD). Figure S1a shows that the sharp characteristic peak (002) of the diffractogram is moved to a lower angle, and the peak of Ti_3_AlC_2_ at 2θ = 39° disappears. This indicates that the Al layer was removed and the Ti_3_AlC_2_ MAX phase etching was successful in forming the Ti_3_C_2_T_x_ crystal structure [46,47]. The XRD patterns of Cu-N/Ti_3_C_2_T_x_ and Cu-Ti_3_C_2_T_x_ (Figure S1b) show a similar crystal structure to Ti_3_C_2_T_x_, with no corresponding peaks in Cu-associated microcrystals observed, suggesting that the Cu species are well dispersed. The surface strain of Cu-N/Ti_3_C_2_T_x_ was observed by using FESEM. And Figure 1b shows the 3D crumpled structure after the introduction of strain, which is favorable to provide more active sites along with improved electronic conductivity. The HAADF-STEAM images confirmed the presence of Cu nanoclusters dispersed on the Ti_3_C_2_T_x_ (Figure 1c). Cu nanoclusters show sharp bright spots because Cu has a larger atomic number than Ti. Also, the lattice spacing of the Cu nanoclusters on Ti_3_C_2_T_x_ is 0.21 nm, which is attributed to the (111) face of metallic Cu [48]. EDS images (Figure 1d,e) showed that Cu and N elements were homogeneously distributed on the Ti_3_C_2_T_x_ substrate. The Cu loading of 3.0 and 2.6 wt% for Cu-N/Ti_3_C_2_T_x_ and Cu-Ti_3_C_2_T_x_, respectively, as measured by ICP-AES, was attributed to the introduction of surface strain to expose more active sites.

**Figure 2:**
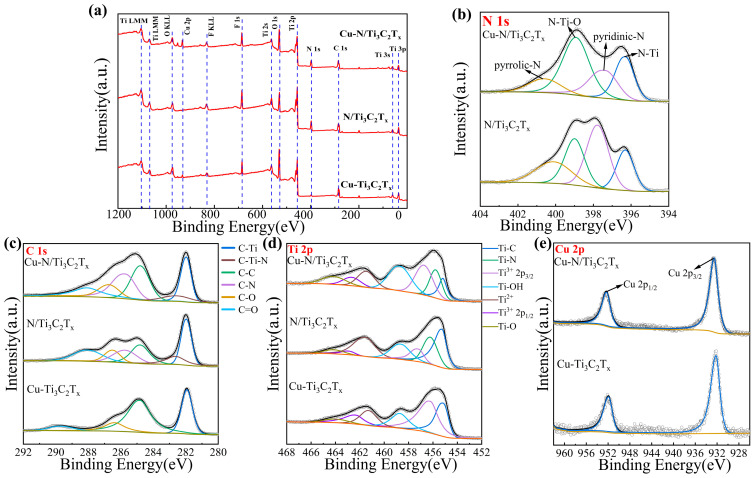
XPS analysis results of Cu-N/Ti_3_C_2_T_x_, N/Ti_3_C_2_T_x_, and Cu-Ti_3_C_2_T_x_. (**a**) XPS survey spectra of Cu-N/Ti_3_C_2_T_x_, N/Ti_3_C_2_T_x_, and Cu/Ti_3_C_2_T_x_. (**b**) N 1s high-resolution XPS spectra. (**c**) C 1s high-resolution XPS spectra. (**d**) Ti 2p high-resolution XPS spectra. (**e**) Cu 2p high-resolution XPS spectra.

The elemental composition and structure of the three synthetic materials were further investigated through XPS characterization. As shown in Figure 2a, the survey spectra of XPS show the presence of C, N, Ti, and Cu, indicating that Cu and N are doped onto the Ti_3_C_2_T_x_ MXene. Figure 2b shows the N 1s spectra of Cu-N/Ti_3_C_2_T_x_ and N/Ti_3_C_2_T_x_, revealing pyridinic N and pyrrolic N, which are two exemplary N-doped structures [49]. Due to the interaction between Cu nanoclusters and lattice N resulting in a positive shift in binding energy, pyrrolic N of Cu-N/Ti_3_C_2_T_x_ (400.6 eV) is 0.5 eV larger than pyrrolic N of N/Ti_3_C_2_T_x_ (400.1 eV) [50]. And the binding energy peaks at 396.3 eV and 398.9 eV correspond to N-Ti and N-Ti-O bonds, respectively, demonstrating the successful substitution of N for lattice carbon. The C 1s spectra of Cu-N/Ti_3_C_2_T_x_, N/Ti_3_C_2_T_x_, and Cu-Ti_3_C_2_T_x_ are shown in Figure 2c. The binding energy peaks of C-Ti, C=C, C-O, and C=O bonds of Cu-Ti_3_C_2_T_x_ are 281.9, 284.8, 286.4, and 289.7 eV, respectively. The formation of C-Ti-N bonds (282.6 eV) and C-N bonds (285.7 eV) results in two more characteristic peaks for Cu-N/Ti_3_C_2_T_x_ and N/Ti_3_C_2_T_x_ than for Cu-Ti_3_C_2_T_x_ [51]. The O 1s spectra (Figure S2) indicate that the binding energy peaks of the C-Ti-O_x_ bond and the Ti-OH bond are 530.1 and 531.6 eV, respectively [45]. Figure 2d shows the Ti 2p spectra of Cu-N/Ti_3_C_2_T_x_, revealing the binding energy peaks of the Ti-C bond (455.2 eV), Ti^3+^ 2p_3/2_ (456.7 eV), Ti^3+^ 2p_1/2_ (462.7 eV), the Ti-OH bond (458.7 eV), Ti^2+^ (461.5 eV), and the Ti-O bond (464.1 eV) [52]. Furthermore, the additional binding energy peak of 455.8 eV compared to Cu-Ti_3_C_2_T_x_ is attributed to the Ti-N bond, which corresponds to the results observed in the N 1s spectra. The Cu 2p spectra of Cu-N/Ti_3_C_2_T_x_ and Cu-Ti_3_C_2_T_x_ (Figure 2e) detected Cu 2p_3/2_ (932.5 eV) and Cu 2p_1/2_ (952.3) peaks, which are attributed to metallic Cu (Cu^0^), demonstrating the successful anchoring of Cu nanoclusters [53]. Meanwhile, the characterization results can prove the absence of CuO, as there are no obvious Cu^2+^ satellite peaks between the binding energy peaks 940 and 950 eV [50].

### 3.2. Analysis of Electrochemical Sensing Responses to DA and UA

**Figure 3:**
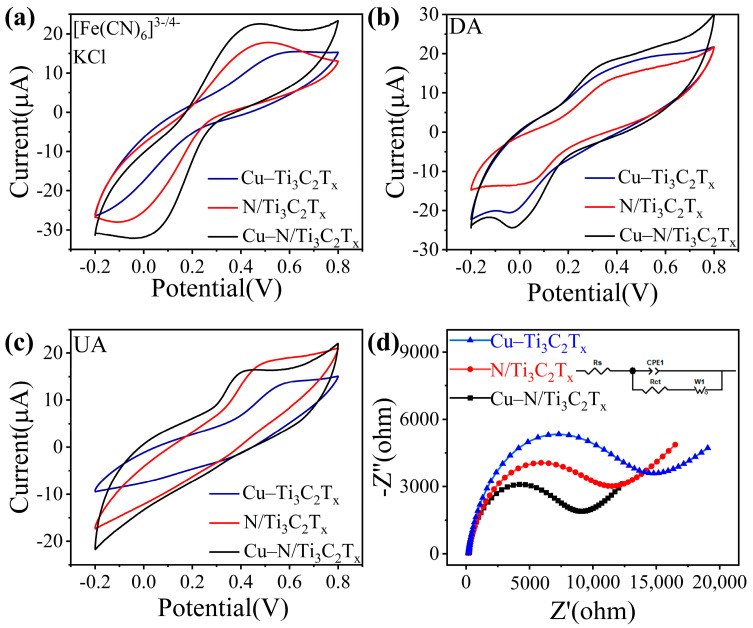
Electrochemical responses. CV curves of Cu-N/Ti_3_C_2_T_x_, N/Ti_3_C_2_T_x_, and Cu-Ti_3_C_2_T_x_ in 0.1 M KCl with (**a**) 5 mM [Fe(CN)_6_]^3−/4−^, and in PBS (pH = 7.4, 0.1 M) with (**b**) 0.5 mM DA, (**c**) 0.5 mM UA. (**d**) The Nyquist plots of EIS at Cu-N/Ti_3_C_2_T_x_, N/Ti_3_C_2_T_x_, and Cu-Ti_3_C_2_T_x_ in 0.1 M KCl with 5 mM [Fe(CN)_6_]^3−/4−^ electrolyte.

Firstly, the CV responses of Cu-N/Ti_3_C_2_T_x_, Cu-Ti_3_C_2_T_x_, and N/Ti_3_C_2_T_x_ modified electrodes were tested in 5 mM [Fe(CN)_6_]^3-/4-^ and 0.1 M KCl solutions. As shown in Figure 3a, the intensities of peak currents exhibited the following trend: Cu-N/Ti_3_C_2_T_x_ > N/Ti_3_C_2_T_x_ > Cu-Ti_3_C_2_T_x_. Due to the doping of Cu nanoclusters and more active sites exposed by the surface strain-modulated 3D crumpled structure, Cu-N/Ti_3_C_2_T_x_ had higher peak currents, suggesting improved electrochemical activity. The effective active area of its electrochemistry was calculated based on the CV curves (Figure S3) with variable sweep speed and the Randles-Sevick equation:

(1) $$I_{P}=2.69\times 10^{5}{AD}^{1/2}n^{3/2}\gamma^{1/2}C$$

where A is the calculated electrochemical active area (cm^2^) of the electrode, I_p_ and γ are the peak current and scan rate of the CV curves in Figure S3, and the parameters D, n, and C are the diffusion coefficient of the redox probe (6.7 × 10^−6^ cm^2^·s^−1^), the number of electrons transferred during the reaction, and the concentration of the redox probe (5 × 10^−6^ mol·cm^−3^) [54]. Subsequently, the three Cu-N/Ti_3_C_2_T_x_, Cu-Ti_3_C_2_T_x_, and N/Ti_3_C_2_T_x_ modified electrodes were compared by CV response in phosphate-buffered saline (PBS) (pH = 7.4, 0.1 M) containing 0.5 mM DA and 0.5 mM UA. The redox mechanisms of DA and UA are as follows: the redox reaction of DA exhibits the loss of 2 H^+^ and 2 e^−^ from dopamine to produce o-dopaminoquinone; the loss of 2 H^+^ and 2 e^−^ during the oxidation of UA results in its oxidation to dehydroxyuric acid. In Figure 3b, the CV response currents of Cu-N/Ti_3_C_2_T_x_ were higher than those of the other two, which indicated that Cu nanoclusters anchored to N-doped crumpled MXene have the best promotion effect on DA catalysis. The oxidation peak currents of Cu-N/Ti_3_C_2_T_x_ and N/Ti_3_C_2_T_x_ in Figure 3c were significantly higher than those of Cu-Ti_3_C_2_T_x_, suggesting that N doping contributed to the enhancement of the electrochemical performance of MXene [55,56]. Cu-N/Ti_3_C_2_T_x_ exhibited superior catalytic performance for DA and UA, attributed to the anchoring of Cu nanoclusters and the N doping to enhance the electrochemical performance. Next, the relationship between the redox peak currents of Cu-N/Ti_3_C_2_T_x_ in DA and UA and scan rate was explored, as shown in Figure S4a,c, which show the CV response curves for a gradient change in scan rate from 10 to 100 mV/s. Figure S4b,d show the linear relationship between the peak current and the scan rate for DA and UA, respectively, indicating that their electrochemical sensing is a diffusion-controlled process [57,58]. Electrochemical impedance spectroscopy (EIS) can effectively represent the electron transfer process at the electrode surface. Figure 3d shows the Nyquist curves of the three modified electrodes in 0.1 M KCl with 5 mM [Fe(CN)_6_]^3−/4−^. By analyzing the equivalent circuit fitting (shown in the inset of Figure 3d), the interfacial charge transfer resistance (R_ct_) can be obtained as 5345 Ω (Cu-N/Ti_3_C_2_T_x_), 9192 Ω (N/Ti_3_C_2_T_x_), and 10,655 Ω (Cu-Ti_3_C_2_T_x_). Figure S5a,b show the Nyquist plots in DA and UA, respectively, where Cu-N/Ti_3_C_2_T_x_ also has the smallest interfacial transfer resistance (3895 Ω for DA, 5579 Ω for UA). In comparison to the other two electrodes, the R_ct_ values of Cu-N/Ti_3_C_2_T_x_ showed significantly lower R_ct_ values, while the ones in EIS showed smaller semicircle diameters, indicating better electron transfer rate and electrocatalytic activity. The results show that the enhanced electrochemical sensing of DA and UA by Cu-N/Ti_3_C_2_T_x_ may be attributed to the fact that, on the one hand, the doping of Cu nanoclusters plays a significant role in enhancing the electrocatalytic activity. On the other hand, the strain modulation suggests that the 3D crumpled structure can provide richer active sites.

### 3.3. Analysis of Electrochemical Sensing Performance to DA and UA

**Figure 4:**
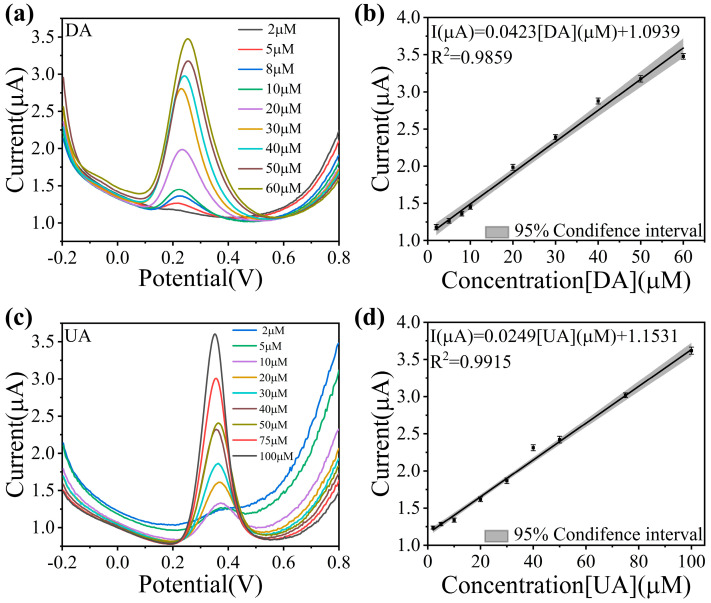
Electrochemical sensing performance. (**a**) DPV curves for Cu-N/Ti_3_C_2_T_x_ at different concentrations of DA. (**b**) The linear relationship between the peak current responses and the DA concentration. (**c**) DPV curves for Cu-N/Ti_3_C_2_T_x_ at different concentrations of UA. (**d**) The linear relationship between the peak current responses and the UA concentration.

Previous tests have demonstrated the excellent performance of Cu-N/Ti_3_C_2_T_x_. To further investigate its sensing and detection ability for DA and UA, the response of Cu-N/Ti_3_C_2_T_x_ to different concentrations of DA and UA was investigated by CV and DPV. The results for DA were shown in Figure 4a and Figure S6a. The peak currents increased with the increase in DA concentration, with a good linear relationship between the two (Figure 4b). The gray area in Figure 4b represents the 95% confidence interval, indicating that there is a 95% probability that the true average peak response current falls within this range for a given concentration, supporting the reliability of the linear model. Based on the results of the DPV test, the linear correlation equation was plotted in the calibration graph:

(2) $$I\left(\mu A\right)=0.0423C_{\left[DA\right]}\left(\mu M\right)+1.0939\left(R^{2}=0.9859\right)$$

The same excellent sensing performance is present in the UA tests shown in Figure 4c and Figure S6b, with a good linearity of the peak currents in the range of 5–100 μM (Figure 4d) and a linear correlation equation of:

(3) $$I\left(\mu A\right)=0.0249C_{\left[UA\right]}\left(\mu M\right)+1.1531\left(R^{2}=0.9915\right)$$

Taking into account the above experimental results and data, the Cu-N/Ti_3_C_2_T_x_ modified electrode had good detection performance for DA and UA with highly sensitive response (1958.3 and 1152.7 μA·mM^−1^·cm^−2^ for DA and UA) and very low detection limit (0.0584 and 0.0993 μM for DA and UA). In comparison with other analog sensors, the Cu-N/Ti_3_C_2_T_x_ exhibits very low detection limits for detecting DA and UA (Table S1).

**Figure 5:**
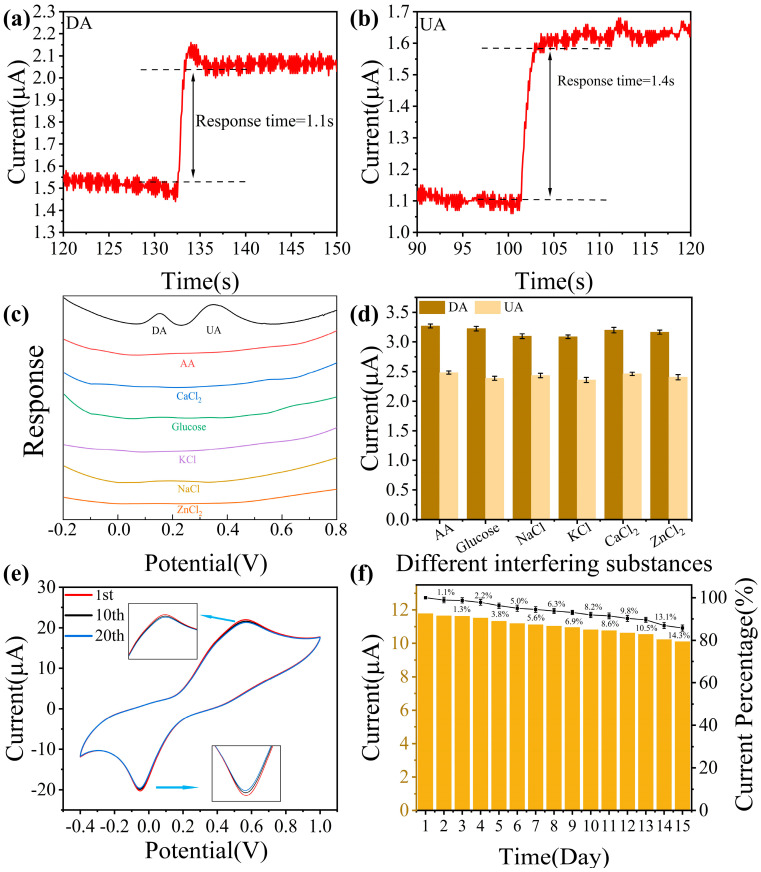
Response time, selectivity, repeatability, and stability of electrochemical sensing. Instantaneous response time of Cu-N/Ti_3_C_2_T_x_ to (**a**) DA and (**b**) UA. (**c**) DPV responses of Cu-N/Ti_3_C_2_T_x_ for DA, UA, and other interferences. (**d**) Influence of multiple interfering substances on the effects of simultaneous detection of 50 μM DA and UA. (**e**) Stability of Cu-N/Ti_3_C_2_T_x_ after 10 and 20 cycles of scanning in 0.1 mM DA and UA. (**f**) The oxidation peak current on the same Cu-N/Ti_3_C_2_T_x_ at 15 days of storage at room temperature.

The fast response time is an important indicator of the sensor detection performance, which requires the sensor to have an extremely high sensitivity to the active substance. The I-t transient tests for DA and UA are shown in Figure 5a,b. The Cu-N/Ti_3_C_2_T_x_ reaches 95% of the steady-state current within 1.1 s and 1.4 s, respectively, indicating high transient responsiveness to DA and UA. Interference immunity is also a key capability for sensor performance testing. The strong adsorption and oxidizing activity of ascorbic acid (AA) are common sources of interference in electrochemical sensors. The high concentration of glucose in body fluids tends to lead to nonspecific adsorption. Na^+^ and K^+^ may alter the structure of the bilayer by electrostatic interaction, affecting the efficiency of electron transfer. Divalent Ca^2+^ and Zn^2+^ have a stronger charge shielding effect and may exacerbate charge rearrangement on the sensor surface. As shown in Figure 5c, a comparison of the DPV response of the Cu-N/Ti_3_C_2_T_x_ modified electrode in CaCl_2_, KCl, NaCl, ZnCl_2_, AA, and glucose verified that there was no response current to interfering substances. Figure 5d shows the DPV response currents of the sensor to DA and UA in the presence of different interfering substances, with RSDs of 2.31% (DA) and 1.96% (UA), which proves that the Cu-N/Ti_3_C_2_T_x_ has an extremely excellent anti-interference capability. Figure 5e shows the CV curves of the same Cu-N/Ti_3_C_2_T_x_ modified electrode cycled for 10 and 20 times, and the RSDs of the oxidation-reduction currents were 1.58% and 1.66%, respectively, confirming that Cu-N/Ti_3_C_2_T_x_ has good repeatability. Cu-N/Ti_3_C_2_T_x_ shows excellent reproducibility in Figure S7: the six modified electrodes were tested in 50 μM DA and 100 μM UA. In addition, stability is a key indicator of the sensor’s performance. To investigate the stability of Cu-N/Ti_3_C_2_T_x_, the prepared electrodes were stored at room temperature for 15 days in total. During this period, the peak current under the same conditions was recorded every day. The results are shown in Figure 5f; the electrode still maintains about 94.4% of the sensing performance after 7 days, but the sensing performance decreases significantly after the 10th day, and the current attenuation reaches 14.3% by the 15th day of the test. The sensor maintains excellent stability over a period of 7 days. In addition, a cross-concentration control experiment was designed in order to test the simultaneous synchronization of the DA and UA detection. As shown in Figure S8, the oxidation peak current of DA showed a significant linear increase with its concentration when the UA concentration was kept static at 50 μM, while the fluctuation of the oxidation peak position response value of UA was small. Conversely, UA showed concentration-dependent enhancement when the DA concentration was kept static at 20 μM. The results showed that the oxidation processes of DA and UA on the electrode surface followed independent electron transfer paths when the two components coexisted, which proved that Cu-N/Ti_3_C_2_T_x_ modified electrode possessed excellent synchronous detection characteristics. Lastly, the performance of Cu-N/Ti_3_C_2_T_x_ was tested in real samples by DPV using the standard spiking recovery method. Human urine samples were spiked with 0.1 M PBS (pH = 7.4) diluted to 1000 times and spiked with known different concentrations (10, 30, and 50 μM) of DA and UA, respectively. The recovery rates for DA and UA calculated from the DPV responses of three independent experiments were 98.2–103.9% and 98.9–101.2%, respectively (Table 1), indicating good feasibility of the detection in real samples.

## 4. Conclusions

In this study, a high-efficiency electrochemical sensor based on Cu nanoclusters anchored on nitrogen-doped crumpled Ti_3_C_2_T_x_ MXene was successfully developed for the parallel detection of DA and UA. The synergistic integration of 3D crumpled architecture and N-doping effectively mitigated MXene restacking, exposed abundant active sites, and enhanced ion accessibility, while Cu nanoclusters further amplified electrocatalytic activity by accelerating electron transfer. Structural characterization confirmed the crumpled structure induced by surface strain and the uniform dispersion of Cu nanoclusters (3.0 wt%), which collectively optimized conductivity and surface reactivity. The Cu-N/Ti_3_C_2_T_x_ sensor demonstrated remarkable dual-analyte detection capabilities, achieving ultrahigh sensitivity (1958.3 and 1152.7 μA·mM⁻^1^·cm⁻^2^ for DA and UA, respectively), ultralow detection limits (0.058 and 0.099 μM), and rapid response times (<1.5 s). Crucially, the sensor exhibited exceptional selectivity against common interferents (e.g., ascorbic acid) and maintained 94.4% stability after 7 days, underscoring its robustness for practical applications. Differential pulse voltammetry validated independent linear detection ranges (2~60 μM for DA, 5~100 μM for UA), enabling accurate quantification in complex biofluids. This work advances the design of MXene-based nanocomposites for biomolecular sensing and provides a simple and convenient non-enzymatic platform for real-time monitoring of DA and UA.

## Figures and Tables

**Table 1:** Cu-N/Ti_3_C_2_T_x_ sensor results for spiked DA and UA in human urine samples.

Analyte	Concentration Added (µM)	Found (µM) ^a^	Recovery (%)	RSD (%)
DA	10	10.39	103.9	3.27
	30	29.45	98.2	2.41
	50	51.19	102.4	3.68
UA	10	10.12	101.2	1.92
	30	29.71	99.1	4.01
	50	49.46	98.9	1.88

^a^ Mean of three independent experiments.

## Data Availability

Data are contained within the article.

## Supplementary Materials

The following supporting information can be downloaded at https://www.mdpi.com/article/10.3390/s25082508/s1: Figure S1: XRD patterns of different materials, Figure S2: O 1s high-resolution XPS spectra, Figure S3: Relationship between scan rate and peak oxidation current for Cu-N/Ti_3_C_2_T_x_ in 0.1 M KCl and 5 mM [Fe(CN)_6_]^3−/4−^, Figure S4: Relationship between scan rate and peak current for Cu-N/Ti_3_C_2_T_x_ in DA and UA, Figure S5: The Nyquist plots of EIS in DA and UA, Figure S6: CV curves for Cu-N/Ti_3_C_2_T_x_ at different concentrations of DA and UA, Figure S7: Response currents of Cu-N/Ti_3_C_2_T_x_ modified six electrodes, Figure S8: Simultaneous detection performance of DA and UA by Cu-N/Ti_3_C_2_T_x_, Table S1: Detection performance comparison of previously reported analogues and this work. References [59,60,61,62,63,64,65,66,67].
