# Supplementary material for: Copper Nanoclusters Anchored on Crumpled N-Doped MXene for Ultra-Sensitive Electrochemical Sensing
Source: Sensors (Basel). 2025 Apr 16;25(8):2508. doi: 10.3390/s25082508 (PMC12031129; doi:10.3390/s25082508)
Supplement: Supplementary file 1 [file sensors-25-02508-s001.zip › Suplementary Information.pdf]

Supplementary Material

Copper Nanoclusters Anchored on Crumpled N-Doped $Ti_3C_2T_x$ MXene for Ultra-Sensitive Electrochemical Sensing

Hanxue Yang [1, 2, 3], Chao Rong [1, 2, 3], Shundong Ge [1, 2, 3], Tao Wang[1, 2, 3, *], Bowei Zhang [1, 2, 3, *] & Fu-Zhen Xuan [1, 2, 3, *]

1. Shanghai Key Laboratory of Intelligent Sensing and Detection Technology, East China University of Science and Technology, Shanghai 200237, P.R. China.
2. Key Laboratory of Pressure Systems and Safety of Ministry of Education, East China University of Science and Technology, Shanghai 200237, P.R. China.
3. School of Mechanical and Power Engineering, East China University of Science and Technology, Shanghai 200237, P.R. China.

*Corresponding: T. W. (wangtao@ecust.edu.cn), B. Z. (boweiz@ecust.edu.cn), and F.-Z. X. (fzxuan@ecust.edu.cn)

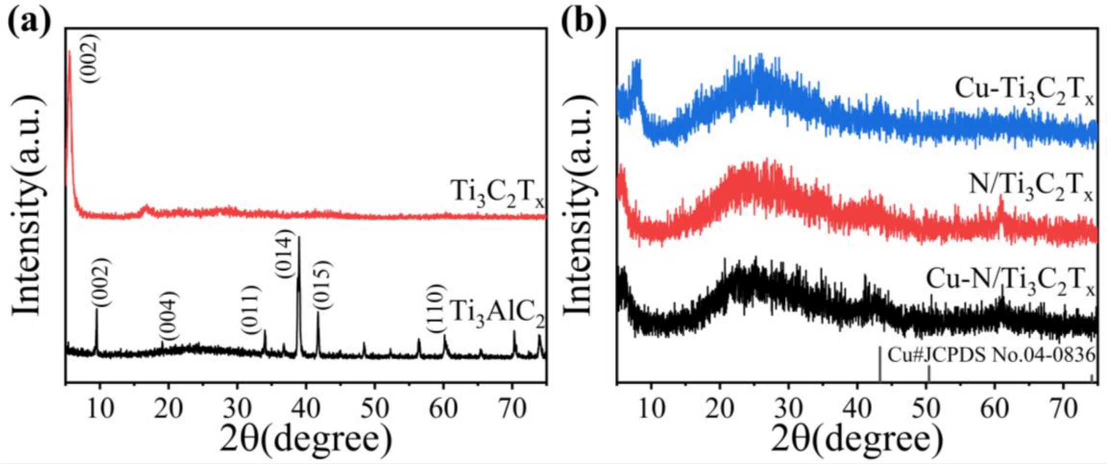

Figure S1. (a) XRD patterns of $Ti_3C_2T_x$ MXene and Ti_3AlC_2 MAX, demonstrating the successful conversion of MAX to MXene. (b) XRD pattern of $Cu\text{-}N/Ti_3C_2T_x$. XRD pattern reveal the atomic dispersion of Cu species.

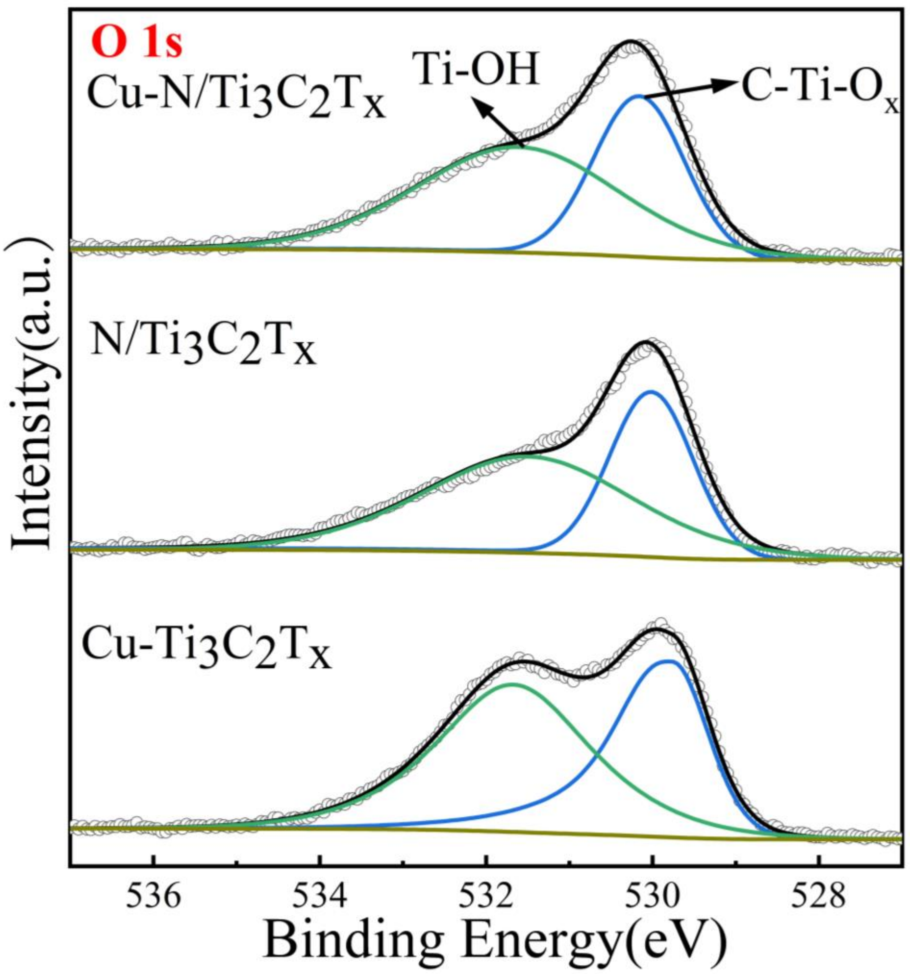

Figure S2. O 1s high-resolution XPS spectra of Cu-N/$Ti_3C_2T_x$, N/$Ti_3C_2T_x$ and Cu-$Ti_3C_2T_x$.

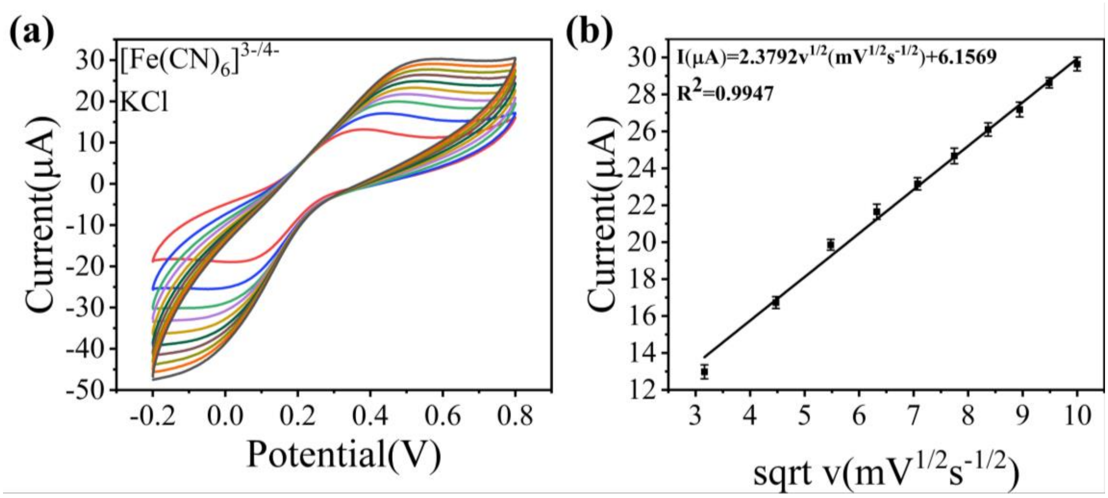

Figure S3. (a) CV curves of Cu-N/$Ti_3C_2T_x$ in 0.1 M KCl with 5 mM $[Fe(CN)_6]^{3-/4-}$ at various scan rates (10-100 mV·s^{-1}). (b) The linear relationship between the peak oxidation current and the square root of the scan rates in in 0.1 M KCl with 5 mM $[Fe(CN)_6]^{3-/4-}$.

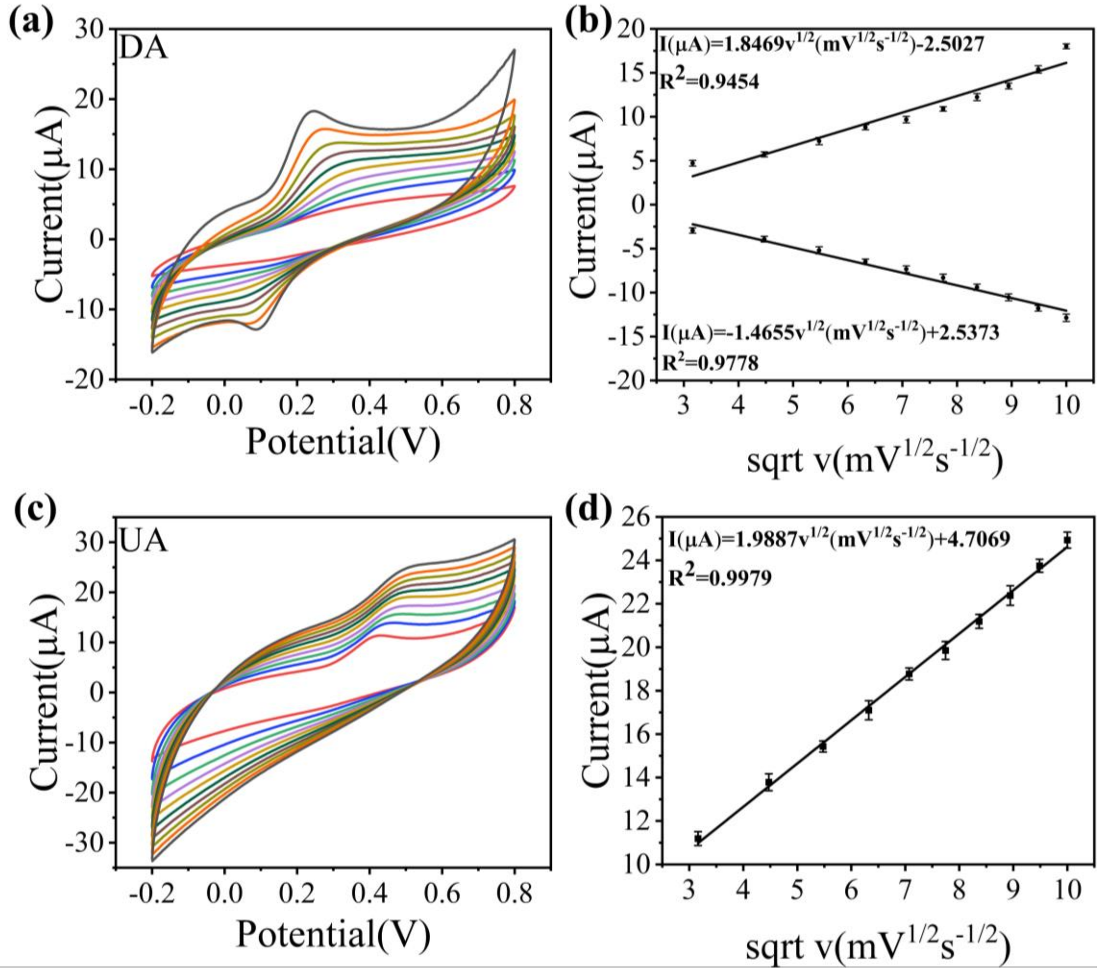

Figure S4. (a) CV curves of Cu-N/$Ti_3C_2T_x$ at various scan rates (10-100 mV·s^{-1}) in DA at a concentration of 200 μM. (b) The linear relationship between the peak oxidation current and the square root of the scan rates in DA solution. (c) CV curves of Cu-N/$Ti_3C_2T_x$ at various scan rates (10-100 mV·s^{-1}) in UA at a concentration of 200 μM. (d) The linear relationship between the peak oxidation current and the square root of the scan rates in UA solution.

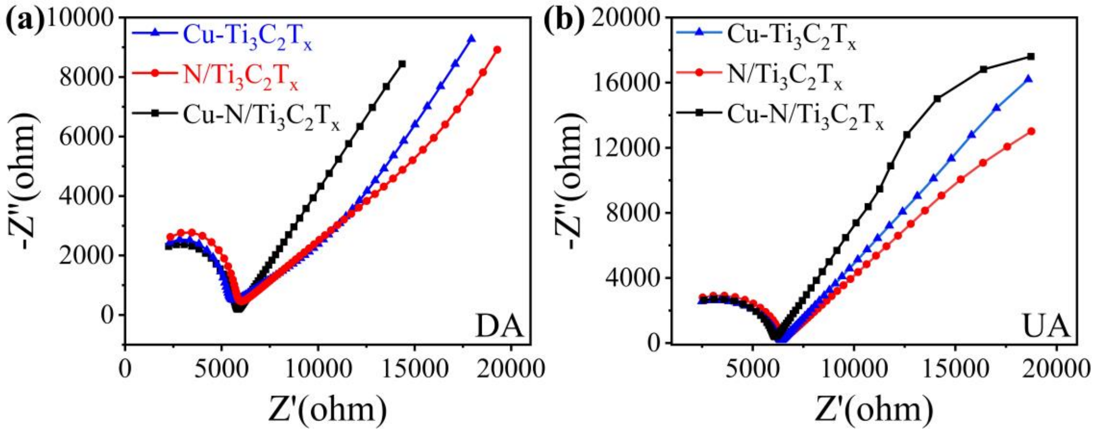

Figure S5. The Nyquist plots of EIS at Cu-N/$Ti_3C_2T_x$, N/$Ti_3C_2T_x$ and Cu-$Ti_3C_2T_x$ in PBS (pH = 7.4, 0.1M) with (a) 0.5 mM DA and (b) 0.5 mM UA.

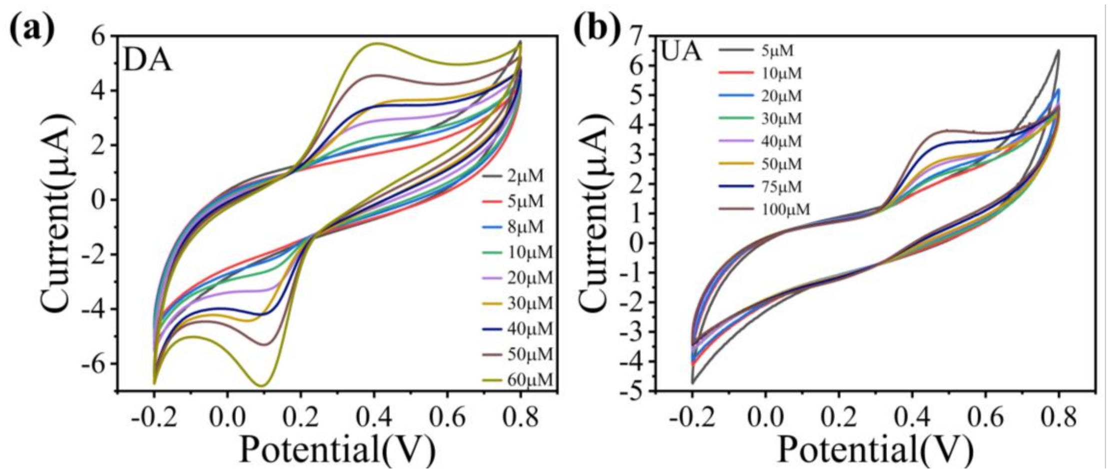

Figure S6. (a) CV curves for Cu-N/$Ti_3C_2T_x$ at different concentrations of DA. (b) CV curves for Cu-N/$Ti_3C_2T_x$ at different concentrations of UA.

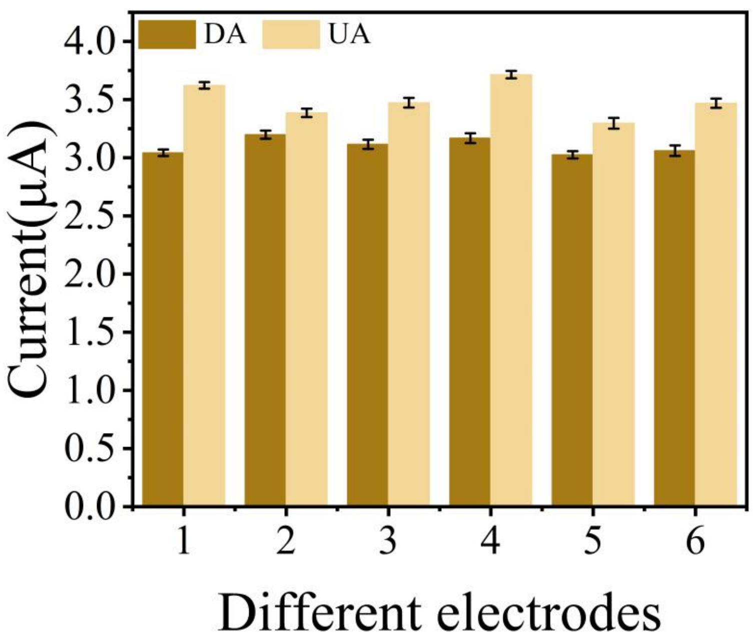

Figure S7. Response currents of Cu-N/$Ti_3C_2T_x$-modified six electrodes in 50 μM DA and 100 μM UA.

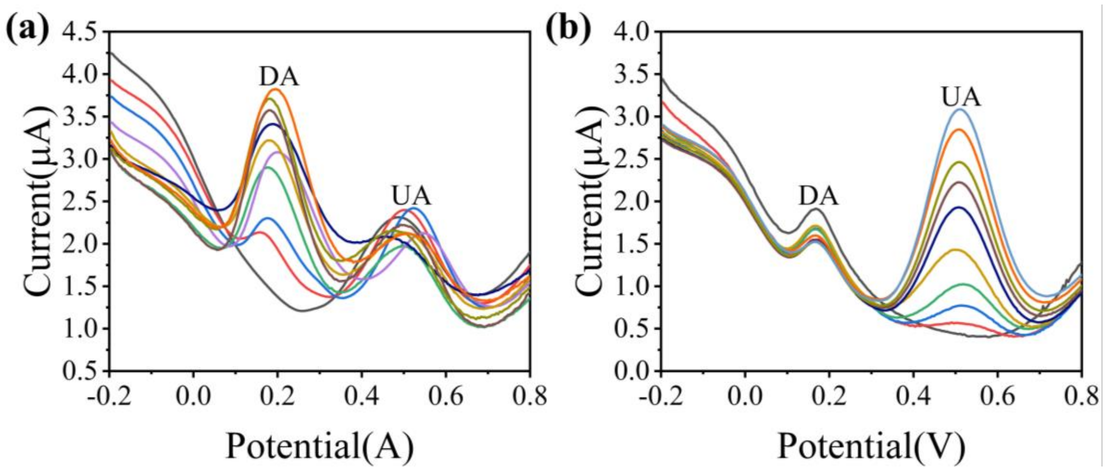

Figure S8. (a) DPV curves for Cu-N/$Ti_3C_2T_x$ at a fixed UA concentration (50 μM) with gradually increasing DA concentration (0 ~ 60 μM). (b) DPV curves for Cu-N/$Ti_3C_2T_x$ at a fixed DA concentration (20 μM) with gradually increasing UA concentration (0 ~ 100 μM).

Table S1. Detection performance comparison of previously reported analogues and this work.

Electrode Materials	Linear range (μM)		Detection limit (μM)		References
	DA	UA	DA	UA	
Pd/MCHS	0.8-50	8-450	0.036	5.00	[1]
MXene/PPy	12.5-125	50-500	0.37	0.15	[2]
Ni-ZIF-8 S-CNTs/CS	8-500	1-600	0.93	0.41	[3]
Au-Pd/MXene/LSG	12-240	8.0-800	0.13	1.47	[4]
3D rGO-Ti_3C_2	0.5-500	0.5-450	0.061	0.085	[5]
Ti-C-T_x	0.5-50	100-1500	0.06	0.075	[6]
Fe-MOF	10-90	10-90	3.33	3.27	[7]
$Ti_3C_2T_x/TiO_2$ NSs	40-300	50-400	0.19	0.25	[8]
3D-GNM	1-30	0.5-100	0.26	6.0	[9]
Cu-N/$Ti_3C_2T_x$	2-60	5-100	0.058	0.099	This work

References

1. Zhang, W.; Li, X.; Liu, X.; Song, K.; Wang, H.; Wang, J.; Li, R.; Liu, S.; Peng, Z. A Novel Electrochemical Sensor Based on Pd Confined Mesoporous Carbon Hollow Nanospheres for the Sensitive Detection of Ascorbic Acid, Dopamine, and Uric Acid. *Molecules* **2024**, *29*, 2427, doi:10.3390/molecules29112427.
2. You, Q.; Guo, Z.; Zhang, R.; Chang, Z.; Ge, M.; Mei, Q.; Dong, W.-F. Simultaneous Recognition of Dopamine and Uric Acid in the Presence of Ascorbic Acid via an Intercalated MXene/PPy Nanocomposite. *Sensors* **2021**, *21*, 3069, doi:10.3390/s21093069.
3. Yao, W.; Guo, H.; Liu, H.; Li, Q.; Wu, N.; Li, L.; Wang, M.; Fan, T.; Yang, W. Highly Electrochemical Performance of Ni-ZIF-8/ N S-CNTs/CS Composite for Simultaneous Determination of Dopamine, Uric Acid and L-Tryptophan. *Microchemical Journal* **2020**, *152*, 104357, doi:10.1016/j.microc.2019.104357.
4. Wang, Y.; Zhao, P.; Gao, B.; Yuan, M.; Yu, J.; Wang, Z.; Chen, X. Self-Reduction of Bimetallic Nanoparticles on Flexible MXene-Graphene Electrodes for Simultaneous Detection of Ascorbic Acid, Dopamine, and Uric Acid. *Microchemical Journal* **2023**, *185*, 108177, doi:10.1016/j.microc.2022.108177.
5. Shang, L.; Li, R.; Li, H.; Yu, S.; Sun, X.; Yu, Y.; Ren, Q. The Simultaneous Detection of Dopamine and Uric Acid In Vivo Based on a 3D Reduced Graphene Oxide–MXene Composite Electrode. *Molecules* **2024**, *29*, 1936, doi:10.3390/molecules29091936.
6. Murugan, N.; Jerome, R.; Preethika, M.; Sundaramurthy, A.; Sundramoorthy, A.K. 2D-Titanium Carbide (MXene) Based Selective Electrochemical Sensor for Simultaneous Detection of Ascorbic Acid, Dopamine and Uric Acid. *Journal of Materials Science & Technology* **2021**, *72*, 122–131, doi:10.1016/j.jmst.2020.07.037.
7. Liu, K.; Chen, Y.; Dong, X.; Huang, H. Simultaneous Voltammetric Determination of Dopamine and Uric Acid Based on MOF-235 Nanocomposite. *Inorganic Chemistry Communications* **2022**, *142*, 109584, doi:10.1016/j.inoche.2022.109584.
8. Jia, D.; Yang, T.; Wang, K.; Wang, H.; Wang, E.; Chou, K.-C.; Hou, X. $Ti_3C_2T_x$ Coated with TiO_2 Nanosheets for the Simultaneous Detection of Ascorbic Acid, Dopamine and Uric Acid. *Molecules* **2024**, *29*, 2915, doi:10.3390/molecules29122915.
9. Gong, J.; Tang, H.; Wang, M.; Lin, X.; Wang, K.; Liu, J. Novel Three-Dimensional Graphene Nanomesh Prepared by Facile Electro-Etching for Improved Electroanalytical Performance for Small Biomolecules. *Materials & Design* **2022**, *215*, 110506, doi:10.1016/j.matdes.2022.110506.
